# Fiber manipulation and post-assembly nanobody conjugation for adenoviral vector retargeting through SpyTag-SpyCatcher protein ligation

**DOI:** 10.3389/fmolb.2022.1039324

**Published:** 2022-12-05

**Authors:** Maryam Kadkhodazadeh, Nasir Mohajel, Mahdi Behdani, Kazem Baesi, Behzad Khodaei, Kayhan Azadmanesh, Arash Arashkia

**Affiliations:** ^1^ Department of Molecular Virology, Pasture Institute of Iran, Tehran, Iran; ^2^ Venom and Biotherapeutics Molecules Laboratory, Medical Biotechnology Department, Biotechnology Research Center, Pasteur Institute of Iran, Tehran, Iran; ^3^ Hepatitis and AIDS Department, Pasteur institute of Iran, Tehran, Iran; ^4^ School of Medicine, Tehran University of Medical Sciences, Tehran, Iran

**Keywords:** SpyTag/SpyCatcher, retargeting, adapter, fiber knob, adenovirus (Ad)

## Abstract

For adenoviruses (Ads) to be optimally effective in cancer theranostics, they need to be retargeted toward target cells and lose their natural tropism. Typically, this is accomplished by either engineering fiber proteins and/or employing bispecific adapters, capable of bonding Ad fibers and tumor antigen receptors. This study aimed to present a simple and versatile method for generating Ad-based bionanoparticles specific to target cells, using the SpyTag-SpyCatcher system. The SpyTag peptide was inserted into the HI loop of fiber-knob protein, which could act as a covalent anchoring site for a targeting moiety fused to a truncated SpyCatcher (SpyCatcherΔ) pair. After confirming the presence and functionality of SpyTag on the Ad type-5 (Ad5) fiber knob, an adapter molecule, comprising of SpyCatcherΔ fused to an anti-vascular endothelial growth factor receptor 2 (VEGFR2) nanobody, was recombinantly expressed in *Escherichia coli* and purified before conjugation to fiber-modified Ad5 (fmAd5). After evaluating fmAd5 detargeting from its primary coxsackie and adenovirus receptor (CAR), the nanobody-decorated fmAd5 could be efficiently retargeted to VEGFR2-expressing 293/KDR and human umbilical vein endothelial (HUVEC) cell lines. In conclusion, a plug-and-play platform was described in this study for detargeting and retargeting Ad5 through the SpyTag-SpyCatcher system, which could be potentially applied to generate tailored bionanoparticles for a broad range of specific targets; therefore, it can be introduced as a promising approach in cancer nanotheranostics.

## Introduction

Recently, various nano-sized viral and non-viral vectors (nanovectors) have been employed in cancer theranostics to maximize efficacy, while minimizing the side effects. Considering numerous advantages and disadvantages of the nanovectors (Supplementary Table S1), bionanoparticles can be excellent alternatives to inorganic nanoparticles owing to their higher biocompatibility and biodegradability (Chung et al., 2020). The optimal bionanoparticles for cancer theranostics should be capable of self-assembly, targeting, cell entry, and endosomal escape. Accordingly, viruses have been applied as great naturally occurring nanocarriers for theranostic applications (Somiya et al., 2017).

Among various viral vectors, adenoviruses (Ads) have been extensively used in clinical trials for gene therapy and vaccination due to their high *in vivo* stability and gene transfer efficiency (Ginn et al., 2018; Singh et al., 2019). More importantly, the safety of Ad-based vectors has been advocated in preclinical and clinical trials (Hajeri et al., 2020). Despite the strong safety profile of these vectors, besides remarkable advances in Ad vector-mediated gene therapy, their clinical application remains challenging. The challenges are mainly attributed to the broad tropism of Ad vectors due to the high affinity of Ad fiber-knob domains for cellular receptors, including the widely expressed coxsackie and adenovirus receptors (CARs) (Zhang and Bergelson, 2005). In contrast, cancer cells mainly have low/no expression of native Ad receptors (Okegawa et al., 2001). It is known that the CAR distribution influences the Ad biodistribution *in vivo*; therefore, intravenous administration of Ads may result in liver toxicity owing to the higher rate of liver transduction (Tao et al., 2001). Accordingly, Ad vectors modified with active targeting modalities have been developed to deal with the resistance of tumor cells and non-specific uptake toxicity and to facilitate efficient gene delivery with fewer side effects.

So far, various detargeting and retargeting methods have been proposed and investigated in the literature, including the modification of capsid proteins (e.g., hexon, fiber, and penton) and implementation of bispecific adapter molecules. The majority of previous studies have focused on alterations in the fiber protein, which is a crucial component of capsid, with a significant contribution to Ad tropism. Generally, the fiber protein is a homotrimeric, antenna-shaped protein, which connects with the penton base to generate penton capsomers found at the icosahedral Ad virion vertices (Krasnykh et al., 2000). Ad5 uses a two-step process to penetrate into cells. First, the knob domain of the fiber must connect with the primary CAR on target cells. Second, the penton base makes contact with integrin receptors on the cell surface, triggering viral uptake *via* receptor-mediated endocytosis (Wickham et al., 1993; Tomko et al., 1997).

While genetic modification has been a prosperous approach for virus targeting, successful incorporation of extraneous moieties into capsid proteins requires extensive protein engineering, which is both challenging and time-consuming. Besides, replacement of the capsid fiber protein mainly results in the production of structurally unstable vectors (Noureddini and Curiel, 2005; Waehler et al., 2007). Also, some targeting ligands require post-translational modifications, such as disulfide bonds that are not present in the cytoplasm or nucleoplasm of cells, where the fiber and Ad particle production occurs (Magnusson et al., 2002).

Comparatively, adapter-based systems that can couple various adapters into the same vector are flexible platforms with no impact on the vector structure. They concurrently eliminate native viral tropisms and facilitate a novel tropism toward the desired target (Dmitriev et al., 2000; Pereboev et al., 2004). However, the majority of adapter systems have drawbacks that limit their potential use in theranostics. The most significant disadvantage of adapter systems is the suboptimal stability of the vector-adapter complex because of unanticipated interactions with other elements that interrupt non-covalent binding (Waehler et al., 2007). To address this challenge, methods that can produce Ad vectors capable of binding to other molecules through covalent interactions, without any need for virus engineering, can be effective.

The SpyCatcher/SpyTag system, a protein-peptide pair forming an isopeptide bond when exposed to each other, has been introduced to create universal vectors (Zakeri et al., 2012). This system is based on the immunoglobulin-like collagen adhesion domain (CnaB2) of *Streptococcus pyogenes*, containing an internal isopeptide bond between Lys31 and Asp117 (Oke et al., 2010; Li and Fierer, 2014). This isopeptide bond is stable over a wide range of pH, temperatures, redox environments, and detergents (Dovala et al., 2016). Since its introduction in 2012, the SpyTag-SpyCatcher system has been implemented in various studies, involving bioactive hydrogels (Sun et al., 2014), thermostabilized proteins (Schoene et al., 2014), multivalent antigen-presenting vaccines derived from virus-like particles (Brune et al., 2016), and lentivirus retargeting (Kasaraneni et al., 2017).

The present study aimed to investigate whether Ad tropism can be altered by the SpyTag-SpyCatcher system, resulting in covalent binding between the virus and the targeted adapter molecule. For this purpose, the feasibility of native Ad5 fiber replacement with a recombinant fiber containing the SpyTag peptide was assessed. Besides, the ability of the modified fiber to bind to the SpyCatcher, as well as the ablation of CAR-mediated internalization of virions following bioconjugation with the SpyCatcher, was examined. Subsequently, a retargeted Ad vector was generated as a model using an adapter molecule, which was constructed through the genetic fusion of SpyCatcher with a nanobody specific to vascular endothelial growth factor receptor-2 (VEGFR2), as one of the main targets for the inhibition of tumor angiogenesis.

Based on the results, the recombinant Ad vector with a SpyTag peptide in its HI loop could robustly engage the adapter molecule to target VEGFR2-expressing cells through a CAR-independent cell entry mechanism. According to the findings, this functionalized Ad vector has great potential applications in cancer theranostics. To the best of our knowledge, this is the first study to evaluate Ad functionality following fiber modification *via* insertion of SpyTag into the HI loop and its bioconjugation with the targeting SpyCatcher-containing adapter molecule.

## Materials and methods

### Construction of modified Ad vector

The protocol proposed by Wu and Curiel was used to produce a pFiberShuttle vector, containing the SpyTag peptide within the knob HI loop (Wu and Curiel, 2008). Accordingly, a 3.5-kb fragment from the plasmid pAdeasy-1 (Addgene, Uniteed States), encompassing a fiber-coding gene and homologous recombination (HR) arms, was subcloned in pUC-19 between *Eco*RI-*Kpn*I restriction sites. Subsequently, a 670-bp fragment, incorporating a 13-amino-acid SpyTag coding sequence (AHIVMVDAYKPTK) following amino acid G543 in the HI loop, was ordered to be synthesized by Biomatik (Ontario, Canada). It was then subcloned between *Afl*II-*Bgl*II digestion sites of the 3.5-kb, subcloned fragment to replace the homologous segment of the intact fiber.

Additionally, to generate a fiber-modified Ad5, an E1 and E3-deleted backbone vector, that is, pAdeasy-1, was used. To facilitate recombination, the pFiberShuttle vector and the backbone should be linearized near or in the position of the fiber gene. Accordingly, a unique *Swa*I cut site was introduced into the fiber gene of pAdeasy-1 vector. For this purpose, pAdeasy-1 was first digested with *Bam*HI, and the produced 11,753-bp fragment, containing the fiber gene and a unique *Nde*I restriction site, was subcloned into the pBluescript-SK vector (Addgene, Uniteed States) (Wu and Curiel, 2008). Afterward, a pair of oligonucleotides (Supplementary Table S1) containing the *Swa*I cut site, as well as sticky ends that ligate in the *Nde*I restriction site, were synthesized and inserted into the corresponding *Nde*I cut site of the subcloned, 11,753-bp, fiber-coding fragment. Finally, the intact homologous pAdeasy-1 fragment was replaced with the modified fragment containing the *Swa*I cut site.

**FIGURE 1:**
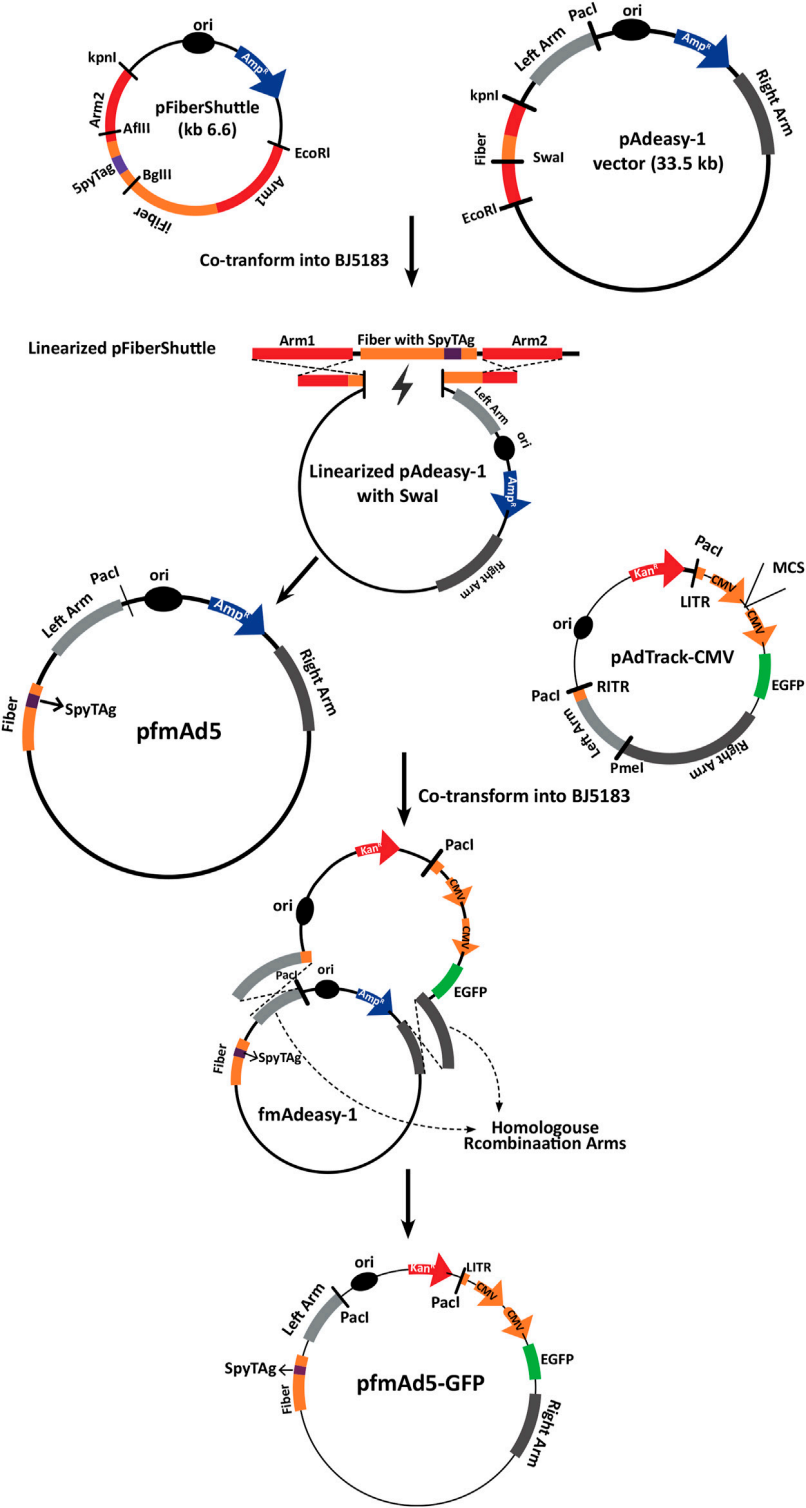
A schematic representation of the development process of pfmAd5-GFP. The pFiberShuttle and pAdeasy-1 constructs were first co-transformed into *E. coli* BJ5183 for a homologous recombination (HR) to generate pAdeasy-1 containing SpyTag. This vector was then used in HR with pAdTrack-CMV containing an *EGFP* gene to create pfmAd5-GFP. The pAd5-GFP, as the control virus, was developed using intact pAdeasy-1 and pAdTrack-CMV for HR.

The fiber-modified Ad backbone vector (pfm-Ad5) was constructed by homologous recombination (HR) between the linearized pFiberShuttle vector and the modified pAdeasy-1 in *E. coli* BJ5183 (Figure 1). Polymerase chain reaction (PCR) with specific primers (Supplementary Table S1) was performed to verify the presence of SpyTag sequence in the Ad5 backbone fiber. The pfm-Ad5 vector was then used to generate the recombinant Ad, encompassing an enhanced green fluorescent protein (EGFP) expression cassette (pfmAd5-GFP) through HR with linearized pAdTrack-CMV vector (Addgene, Uniteed States) (Figure 1).

Additionally, an unmodified Ad5-GFP virus was produced following HR between unchanged pAdeasy-1 and linearized pAdTrack-CMV. To confirm HR, the recombined vectors were extracted and digested with *Pac*I, followed by transfection into the AD-293 cell line, using a Lipofectamine™ 2000 Transfection Reagent (Invitrogen, Uniteed States) to rescue fmAd5-GFP and Ad5-GFP. Following the production of fmAd5-GFP, the presence of SpyTag in the fiber protein was confirmed *via* Ad genome extraction with a QIAamp DSP Virus Kit (Qiagen, Germany), followed by PCR and fiber sequencing. The fmAd5-GFP and Ad5-GFP titers were determined by the Median Tissue Culture Infectious Dose (TCID_50_) assay (Herrmann and Bucksch, 2014).

### Recombinant protein expression

The DNA fragment encoding SpyCatcherΔ, a protein with 21- and 14-amino-acid truncations at the N and C termini of the original protein, respectively (Kasaraneni et al., 2017), was synthesized by Biomatik (Ontario, Canada); it also harbored a 14-amino-acid hinge sequence at its C-terminus. Subsequently, the fragment was subcloned in the pET-28a (+) bacterial expression plasmid (Novagen, Uniteed States) to generate pET28Catcher. Additionally, to generate a SpyTag-expressing plasmid, a pair of oligonucleotides (Supplementary Table S1) containing the SpyTag sequence and sticky ends for ligation into the *Kpn*I and *Sal*I cut sites was synthesized and subcloned into the pET-32a (+) plasmid (Novagen, Uniteed States). The plasmids were then transformed into *E. coli* Rosetta (DE3), and protein expression was induced by the addition of 1 mM isopropyl β-D-1-thiogalactopyranoside (IPTG) for 4 hours at 37°C in a Lysogeny broth (LB) culture medium. The expressed SpyCatcherΔ and TrxA-SpyTag proteins were then purified using Ni-NTA agarose columns (Qiagen, Germany), according to the manufacturer’s protocols.

To evaluate the ability of SpyCatcherΔ to create an isopeptide bond with SpyTag, the purified SpyCatcherΔ (15.8 kDa) was incubated with TrxA-SpyTag (19.4 kDa) at a molar ratio of 1:1 for 1 hour at room temperature. The protein bioconjugation was tested by sodium dodecyl sulfate-polyacrylamide gel electrophoresis (SDS-PAGE). Additionally, to assess the ligation of fmAd5-GFP with the SpyCatcherΔ, 5×10^9^ TCID_50_ of the virus was incubated with 10 µM of the purified SpyCatcherΔ for 2 hours at 37°C. Next, the mixture was denatured by boiling in a sample buffer for 5 minutes at 95°C and subsequently analyzed by SDS-PAGE. The protein was finally blotted onto a polyvinylidene difluoride (PVDF) membrane and developed with ECL Plus Substrate for Western blotting (Thermo Fisher Scientific, Uniteed States) after sequential incubation with a locally-obtained anti-SpyCatcherΔ serum and goat anti-mouse IgG-HRP (Abcam, United Kingdom). The Ad5-GFP was used as the control virus.

To generate an adapter molecule, encompassing a VEGFR2-specific nanobody fused to the SpyCatcherΔ, the nanobody-coding gene was amplified from the p2.2-Nb plasmid (Ahani et al., 2016) and subcloned at the N-terminus of the SpyCatcherΔ-coding sequence in the pET28Catcher plasmid through a flexible SGSGSSGAS linker. The construct was then subcloned in the pHEN6C expression vector containing a C-terminal His6 tag. Next, it was transformed into *E. coli* WK6 cells and induced for protein expression and purification as previously described (Rouet et al., 2012). Finally, the ability of the adapter molecule to covalently bind to the SpyTag was examined by SDS-PAGE.

Moreover, for the Ad5 fiber knob expression, its coding sequence was amplified from pAdEasy-1 (Yang et al., 2006) and subcloned into pET-32a (+). The expression parameters were similar to those of the abovementioned proteins. Nonetheless, due to protein aggregation, phosphate-buffered saline (PBS), containing 2% glycerol and 0.01% Tween 20, was used as dialysis buffer and protein solvent for protein purification.

### Cell lines

In this study, the Chinese hamster ovary cell line (CHO-K1), the human embryonic kidney cell line optimized for Ad propagation (AD-293), the A549 human lung epithelial cell line with a high expression of CARs, the 293/KDR cell line stably overexpressing VEGFR2, and human umbilical vein endothelial cells (HUVEC) as the primary VEGFR2-expressing cell line were used. The cell lines present in this study were obtained from the National Cell Bank of Pasteur Institute of Iran. The AD-293, 293/KDR, and A549 cells were grown in Dulbecco’s Modified Eagles Medium (DMEM, Biosera, Philippines), containing 10% heat-inactivated fetal bovine serum (FBS; Gibco, Uniteed States) and antibiotics (100 U/mL of penicillin and 100 μg/ml of streptomycin) (Biosera, Philippines). The CHO-K1 and HUVEC cells were cultured in DMEM-F12 (Biosera, Philippines), containing 10% FBS and antibiotics as described above.

The CHO-K1 cell line, which stably expresses SpyCatcherΔ on its surface (CHO-Spy), was developed through transfection with a pDisplay plasmid (Thermo Fisher Scientific, Uniteed States), encoding SpyCatcherΔ, according to a previously described protocol (Mortensen et al., 1997). Briefly, before transfection, the susceptibility of CHO-K1 to G418 (BioBasic, Canada) was determined to be 0.4 mg/ml. Transfection was performed with a Lipofectamine 2000 Transfection Reagent. The medium was replaced 48 h after transfection, and fresh DMEM-F12, containing 0.5 mg/ml of G418, was added to the medium. Next, the cells were serially diluted into a 96-well plate and incubated for 14 days to isolate the monoclonal cell line. Twelve monoclonal cells were selected and expanded to analyze the SpyCatcherΔ expression by flow cytometry (CyFlow, Partec, Germany), using the anti-SpyCatcherΔ serum and goat anti-mouse IgG-PE antibody (Thermo Fisher Scientific, Uniteed States).

### Transduction of CHO-K1 and CHO-Spy by Ad vectors

The CHO-K1 and CHO-Spy were cultured at 1.5–2×10^5^ cells/well in 24-well plates and infected with fmAd5-GFP and Ad5-GFP vectors at multiplicity of infection (MOI) of 10, 50, 100, 200, 500, and 1000 TCID_50_/cell for 2 hours. Subsequently, the medium was removed, and 0.5 ml of DMEM/Nutrient Mixture F-12 (DMEM/F-12), containing 2% FBS, was added to each well. The transduction efficiency was evaluated by measuring the fluorescence of cells after 48 h of incubation at 37°C, using fluorescent microscopy and flow cytometry.

### CAR-binding inhibition assay

The A549 cells were first cultured in a 24-well tissue culture plate at a density of 1×10^5^ cells per well. On the following day, fmAd5-GFP, at MOIs of 100 and 400, was combined with 0, 5, 10, and 20 µM of purified SpyCatcherΔ and incubated at 37°C for 2 hours, followed by the addition of SpyCatcherΔ-conjugated virus to each well and incubation for another 2 hours in a cell culture incubator. After the medium removal, 0.5 ml of DMEM, containing 2% FBS, was added to each well. The cells were harvested after 48 h, and the percentage of transduction was measured by flow cytometry.

### Nanobody-conjugated virus transduction

The 293/KDR and HUVEC cells were grown at 1.5–2×10^5^ cells/well in 24-well plates, and their CARs were blocked by the addition of a purified recombinant Ad5-knob protein to each well at a final concentration of 100 μg/ml. Subsequently, the Ad vectors were incubated with 10 µM of SpyCatcherΔ-nanobody adapter molecule and different ratios of the adapter and fiber (1:1, 1:100, and 1:1000) for 2 hours at 37°C. Afterward, fmAd5-GFP and Ad5-GFP (as the control vector) were added at MOIs of 10, 20, and 50 and to the HUVEC cells at MOIs of 100, 200 and 500 TCID_50_/cell. Following 2 hours of incubation at 37°C, the virus-containing medium was withdrawn and replaced with a fresh medium, containing 2% FBS. To prevent reinfection, the 293/KDR cells were cultured for 24 h at 37°C, while the HUVEC cells were cultured for 48 h. Flow cytometry was finally carried out to determine the transduction rate and fluorescence intensity.

## Results

### Generation of fiber-modified Ad vector

A SpyTag-decorated Ad vector, which could covalently bind to the SpyCatcher-fused targeting moiety, was generated, enabling the vector to be retargeted to various ligands, without further genetic modifications of the vector. According to previous studies, considering the crystallographic structure of the Ad fiber, two regions of the knob, that is, the C-terminus and the HI loop, were suitable for incorporating foreign motifs, as they allowed exposure and facilitated viral interaction with the target cell, with unlikely effects on key viral functions (e.g., capsid packaging and viral infection) (Belousova et al., 2002).

For Ad5 retargeting, the SpyTag-coding sequence was inserted into the HI loop of the fiber knob between G543 and D544 residues through HR (Dmitriev et al., 1998). The fmAd5-GFP and Ad5-GFP were rescued and upscaled in AD-293 cells after verifying the presence of SpyTag in the Ad fiber genome by PCR. After viral amplification, the Ad genome was extracted, and the presence of SpyTag sequence was reconfirmed by fiber sequencing (Supplementary Figure S1). To determine whether the insertion of 13-amino-acid peptides affected viral replication and titer, fmAd5-GFP and Ad5-GFP were amplified under similar conditions and titrated using the TCID_50_ assay. Their titers were nearly the same, equivalent to 5×10^10^ TCID_50_/mL.

### Expression and purification of recombinant proteins

The original SpyTag-SpyCatcher system consisted of a 13-amino-acid SpyTag and a 138-amino-acid SpyCatcher (Li and Fierer, 2014). In this study, a modified SpyCatcher was used with 21- and 14-residue truncations at the N and C termini, respectively, as full-length SpyCatcher has been identified to interact with an unknown cell surface receptor, leading to significant background transduction (Kasaraneni et al., 2017). However, before assessing the ability of SpyCatcherΔ to bind to fiber-modified Ad containing SpyTag, its potential to bind to free SpyTag was investigated. For this purpose, SpyCatcherΔ and TrxA-SpyTag were recombinantly expressed in *E. coli* Rosetta (DE3) and purified by exploiting their His-tag for Ni-NTA chromatography.

**FIGURE 2:**
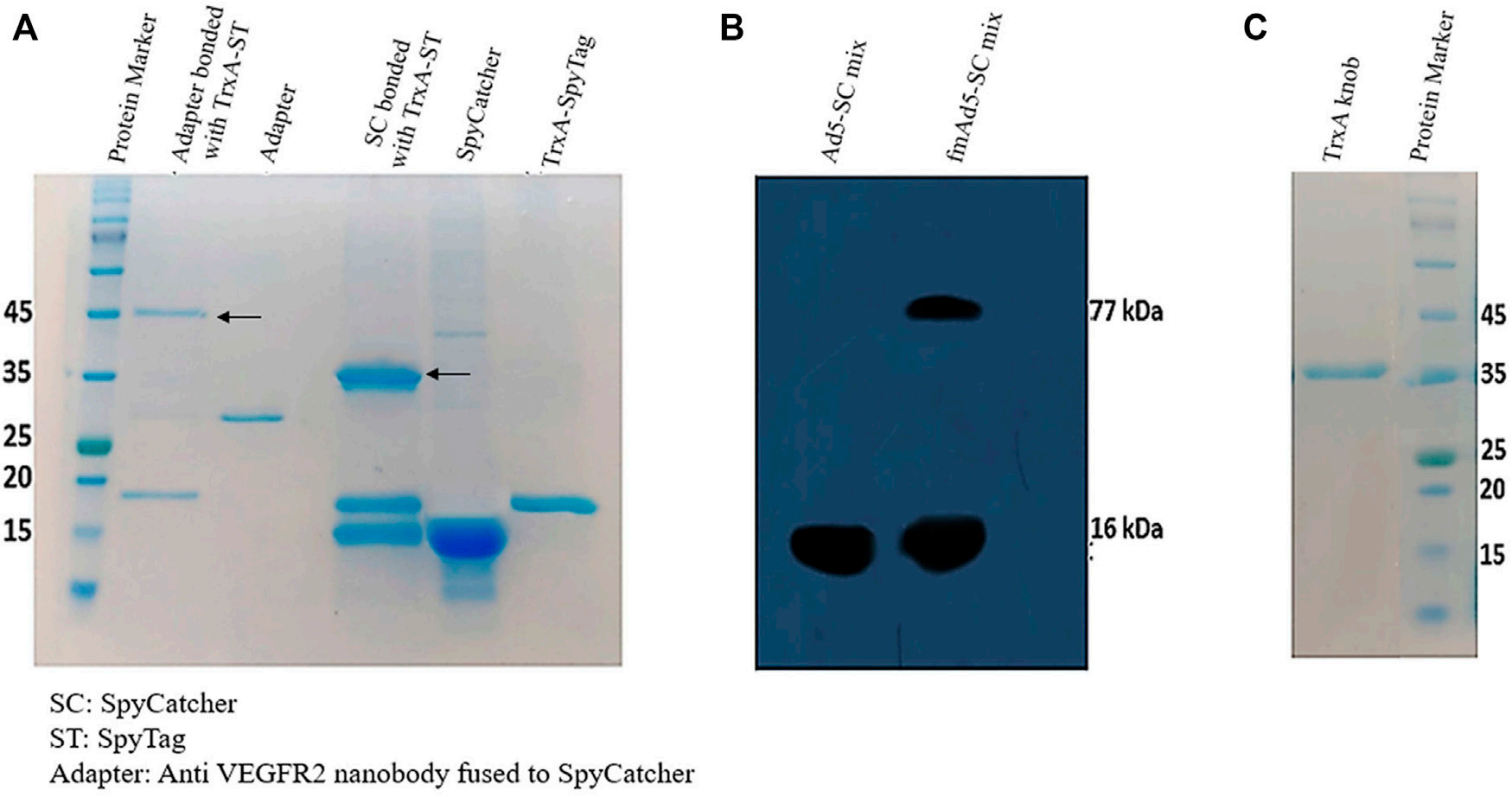
Characterization of protein expression and binding. **(A)** The SDS-PAGE analysis of SpyCatcher-TrxA-SpyTag bioconjugation. The purified TrxA-SpyTag was mixed with SpyCatcher and adapter (SpyCatcher-anti VEGFR-2 nanobody) at a molar ratio of 1:1 at room temperature for 1 hour. **(B)** Western blotting analysis to identify Ad-modified fiber bound to the SpyCatcherΔ. The vector (5 × 10^9^ TCID_50_) was incubated for 2 hours at 37°C with 10 µM of purified SpyCatcherΔ before boiling and application on 12% SDS-PAGE gel, followed by blotting to a PVDF membrane; it was finally probed with a polyclonal antibody against the SpyCatcherΔ and goat anti-mouse IgG (HRP). **(C)** The 12% SDS-PAGE analysis of the purification of recombinant knob protein fused to TrxA and His tags.

The TrxA-SpyTag and SpyCatcherΔ were highly expressed in *E. coli* Rosetta (DE3), yielding 20 mg/L of purified protein. However, no indication of adapter molecule expression was found in *E. coli* Rosetta (DE3) cells using various pET vectors; accordingly, the pHEN6C expression vector and *E. coli* WK6 cells, which were optimized for nanobody production, were employed. The Ni-NTA chromatography was also used to purify the recombinant adapter molecule. Next, the adapter protein, comprising of the anti-VEGFR2 nanobody-SpyCatcherΔ fusion, was combined with the TrxA-SpyTag peptide to examine whether TrxA-SpyTag could bioconjugate with the adapter molecule. The complex formation was assessed using SDS-PAGE. As demonstrated in Figure 2A, the adapter molecule could form a stable linkage with TrxA-SpyTag.

Subsequently, Western blotting was performed to determine the presence of SpyTag in the fiber structure and its capability to bind to its SpyCatcherΔ pair. As shown in Figure 2B, besides the SpyCatcherΔ band (16 kDa), a band of approximately 77 kDa was observed, suggesting the bonding of Ad-modified fiber (61 kDa) with the SpyCatcherΔ. However, when the control virus, Ad5-GFP, was mixed with the SpyCatcherΔ, this 77-kDa band was absent. The Ad5 knob protein was also produced in *E. coli* Rosetta (DE3), with TrxA and His tags at its N-terminus, allowing for single-step isolation using Ni-NTA chromatography, which indicated a single ∼38 kDa band on SDS-PAGE (Figure 2C). Moreover, the functionality of recombinant knob protein was assessed using 293/KDR and AD-293 cell lines. The ability of the protein to block CAR and prevent the internalization of Ad5 is presented in Supplementary Figure S2.

### Establishment of the CHO-Spy cell line

**FIGURE 3:**
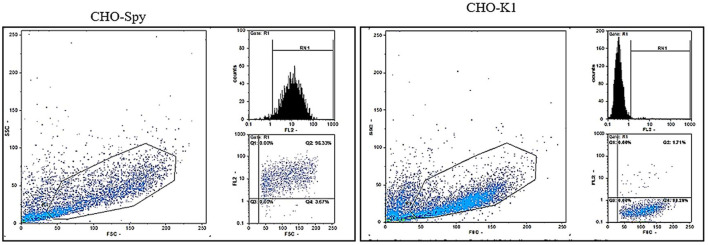
The flow cytometric analysis of SpyCatcherΔ expression after CHO-KI transfection. Following the transfection of CHO-KI cells with the pDisplay plasmid encoding SpyCatcherΔ, 12 clones were isolated, and the level of SpyCatcherΔ expression and homogeneity were determined *via* flow cytometry. Monoclonal cells expressing the SpyCatcherΔ, as well as CHO-KI cells as the negative controls, were treated with the anti-SpyCatcher serum for 1 hour, followed by incubation with the goat anti-mouse Ig-PE. Three out of 12 expanded monoclonal cells demonstrated more than 96% homogeneity and high expression levels of SpyCatcherΔ, as depicted for one of the clones on the left panel. The right panel shows the non-transfected CHO-KI cell line as the negative control.

The CHO-K1 cell line (CAR-negative cells) was transfected with a pDisplay vector, encoding SpyCatcherΔ and neomycin resistance genes to generate a cell line that allowed for the steady expression of SpyCatcher on its surface. Therefore, it was possible to investigate the fmAd5-GFP binding capacity to SpyCatcher on the cell surface, as well as virus internalization *via* SpyCatcher/SpyTag binding. Following G418 selection, the pool of cell clones was expanded, and flow cytometry was performed to evaluate the SpyCatcherΔ expression on the cell surface. Approximately 13% of CHO-K1 cells expressed SpyCatcherΔ. Next, a clonal selection was carried out, yielding 12 monoclonal cells. The SpyCatcherΔ expression level and cell uniformity were also assessed using flow cytometry. Three out of 12 monoclonal cells, which showed the highest expression levels and homogeneity >96%, were finally isolated. Data for one of the selected clones are depicted in Figure 3.

### SpyTag-SpyCatcher-mediated viral vector transduction

**FIGURE 4:**
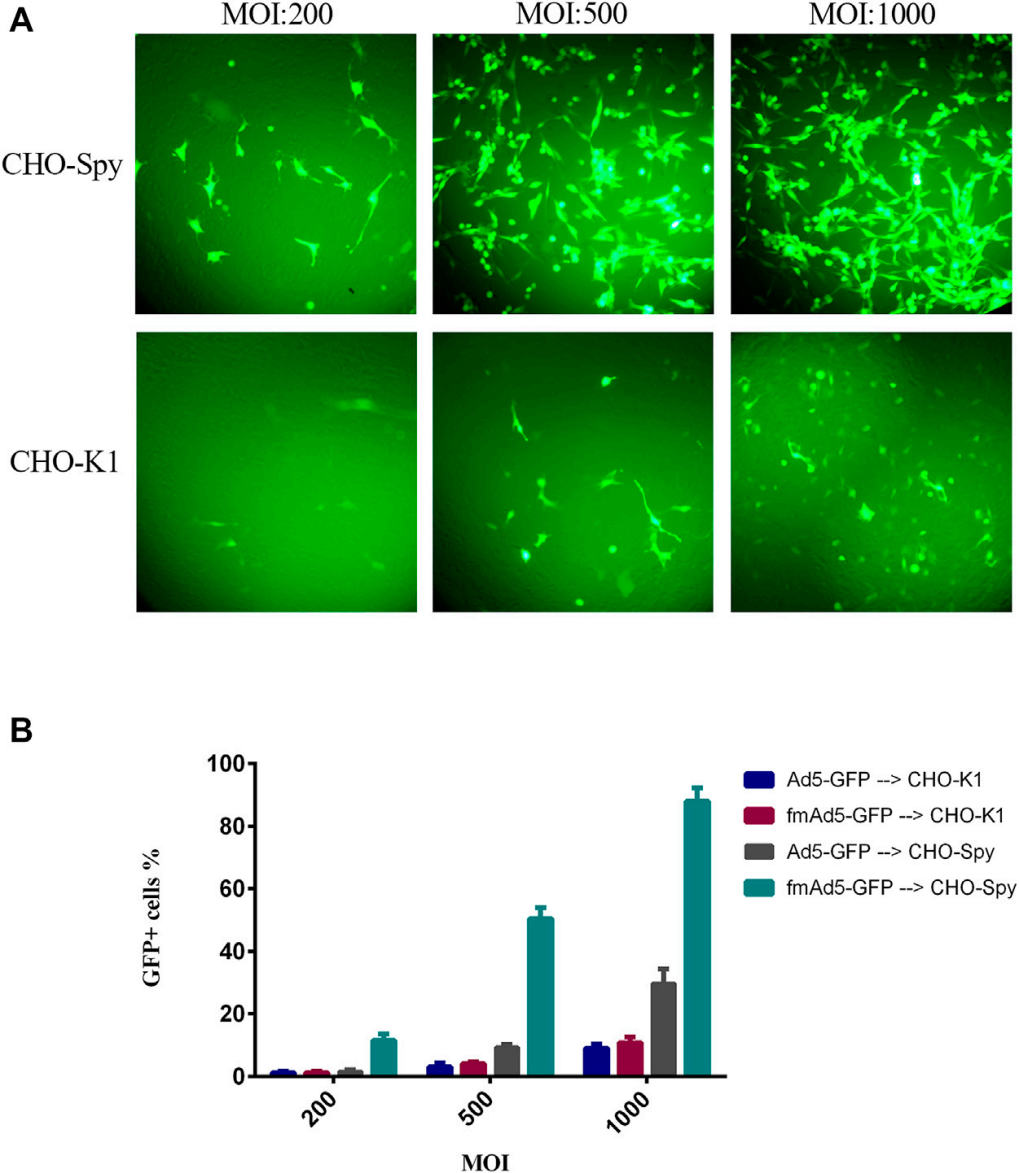
Transduction efficiency of CHO-K1 and CHO-Spy cells with fmAd5-GFP and Ad5-GFP. **(A)** The cells were infected with fiber-modified Ad-GFP at MOIs of 200, 500, and 1000 TCID_50_/cell for 48 h, and the fluorescent signal of EGFP was investigated by fluorescent microscopy. **(B)** The percentage of transducted CHO-K1 and CHO-Spy cells with various MOIs of fmAd5-GFP and Ad5-GFP vectors according to flow cytometry; data are presented based on duplicate experiments, and values are presented as mean ± SEM.

The transduction efficiency of CHO-K1 and CHO-Spy cells was examined with fmAd5-GFP and Ad5-GFP to primarily examine the functionality of the SpyTag-harboring viral vector in binding to the cell surface-expressed SpyCatcher. It should be noted that the CHO-K1 cells do not normally express detectable levels of human CAR; consequently, they are normally non-permissive to natural Ads. The CHO-Spy cells that were able to express SpyCatcherΔ on the cell surface were also developed. The transduction efficiency of both cell lines was negligible at MOIs of 10, 50, and 100 for both viral vectors at 48 h post-infection (data not shown), while at higher MOIs (200, 500, and 1000) of fmAd5-GFP and Ad5-GFP, as shown in Figure 4A, there was a significant difference in transduction efficiency between CHO-K1 and CHO-Spy with fmAd5-GFP at all MOIs. The results were confirmed using flow cytometry, which indicated the percentage of EGFP-positive cells and the mean fluorescence intensity after infection with each vector. In case of both viral vectors, CHO-K1 cells showed the lowest percentage of EGFP-positive cells and the lowest overall fluorescence intensity in the transduced cells, as expected (Figure 4B).

**FIGURE 5:**
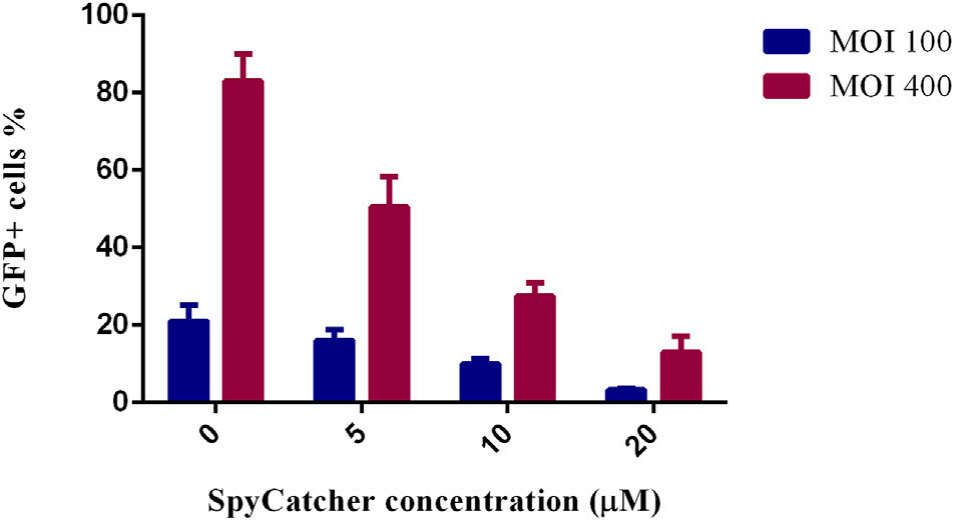
Ablation of CAR-mediated transduction of A549 cells. The percentage of GFP^+^ cells is shown after the incubation of fmAd5-GFP at MOIs of 100 and 400 by increasing the concentration of SpyCatcherΔ before infecting the A549 cells. Data are based on duplicate experiments, and values are represented as mean ± SEM.

On the other hand, when the CHO-Spy cells were infected with the Ad5-GFP control virus, the transduction rate was slightly higher than CHO-K1 cells, but significantly lower than the fmAd5-GFP. It was hypothesized that the slight increase in the Ad5-GFP transduction rate in CHO-Spy cells might be related to the inaccurate measurement of CHO-Spy cells because of their high adhesion capability following the SpyCatcherΔ expression. In the CHO-Spy cells, transduction with fmAd5-GFP at MOI of 1000 resulted in the transduction of nearly 90% of cells *versus* 9% of CHO-K1 cells. Besides, the EGFP fluorescence intensity was twice higher, indicating the efficient transduction of fmAd5-GFP into the CHO-Spy cells through bioconjugation of the Spy-tagged viral vector with cell surface-expressed SpyCatcher. Also, differences of approximately 9–10 folds in transduction (as shown in Figure 4B) and 2-5 folds in fluorescence intensity were identified at other MOIs.

### Blockade of CAR-mediated transduction

This study assessed whether SpyCatcher conjugation to fmAd5-GFP resulted in the ablation of CAR-mediated transduction of A549, as a high CAR-expressing cell line. Accordingly, fmAd5-GFP was incubated at MOIs of 100 and 400 with 0, 5, 10, and 20 µM of SpyCatcherΔ at 37°C for 2 hours before infecting the A549 cells. As shown in Figure 5, the percentage of EGFP-positive cells decreased with an increase in the SpyCatcher protein concentration. When fmAd5-GFP was incubated with 20 µM of SpyCatcher at both MOIs, the number of positive EGFP cells reduced by seven folds relative to the non-SpyCatcher control group; consequently, the SpyCatcherΔ binding to the fmAd5-GFP could effectively reduce the virus entry *via* CARs.

### VEGFR2-expressing cell transduction by the retargeted viral vector

**FIGURE 6:**
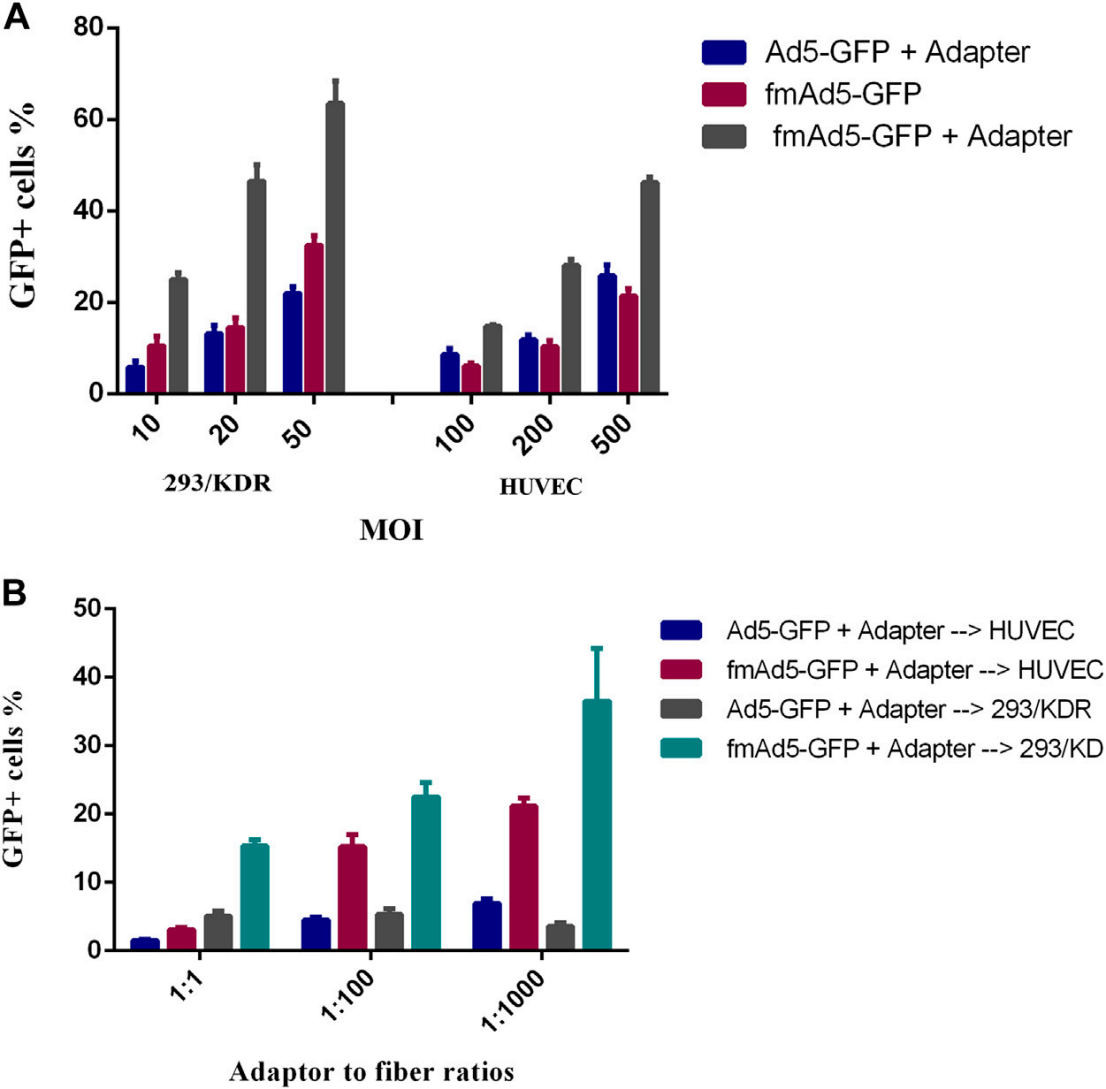
Transduction of VEGFR2-expressing cells with the retargeted adenoviral (Ad) vector. **(A)** Comparison of the transduction efficiency of adapter-mixed Ad5-GFP with adapter-conjugated fmAd5-GFP. After blocking CAR with the recombinant knob protein, the 293/KDR and HUVEC cells were transduced with different MOIs of adapter-treated Ad5-GFP or fmAd5-GFP. **(B)** After incubating the 293/KDR and HUVEC cells with the knob protein, the cells were infected with Ad vectors that were previously incubated with the adapter at various adapter-to-fiber ratios and MOIs of 20 and 200, respectively. Data are presented based on duplicate experiments, and values are presented as mean ± SEM.

To examine the efficacy of Ad vector retargeting, the 293/KDR cell line, expressing a high level of cell-surface VEGFR2, and HUVEC as the primary VEGFR2-expressing cell line, were transduced with fmAd5-GFP, which was previously conjugated with the adapter molecule. When the 293/KDR and HUVEC cells were transduced with the adapter-conjugated fmAd5-GFP and adapter-mixed Ad5-GFP, there was a significant increase in transduction efficiency of both cell lines with the adapter-conjugated fmAd5-GFP. The flow cytometry revealed that the percentage of EGFP^+^ 293/KDR and HUVEC cells in the adapter-conjugated fmAd5-GFP-transducted group was almost three and two folds higher than the control group at all MOIs, respectively (Figure 6A).

Additionally, a serial increase in the adapter-to-fiber ratio from 1:1 to 1:1000 resulted in a progressive rise in the percentage of EGFP^+^ 293/KDR and HUVEC cells infected with adapter-conjugated fmAd5-GFP; conversely, the rate of transduction with adapter-mixed Ad5-GFP was significantly lower and also invariant (Figure 6B). However, in terms of EGFP fluorescence intensity, there was no significant difference between the adapter-conjugated fmAd5-GFP and the control groups. Based on these findings, although Ad5-GFP mixed with an adapter could partially infect VEGFR2-expressing cells, there was a remarkable increase in the transduction efficiency when the adapter-conjugated fmAd5-GFP was used for both VEGFR2-expressing cell lines.

## Discussion

The current study aimed to present a bionanoparticle-based adaptable technique for modifying the natural receptor specificity of Ad5 vector, which can be used to retarget Ad5 for theranostic applications. Although Ad5 is the most common viral vector modified for gene therapy, development of a safe and efficient vector remains challenging in the clinical setting. One of the main restrictions in the clinical administration of Ads is the promiscuous native Ad tropism, which limits its systemic administration, as it can induce toxicity through non-specific uptake, while decreasing the vector bioavailability for the target cells (Waehler, et al., 2007). Therefore, detargeting and retargeting are crucial strategies for improving the efficacy of Ad-mediated tumor theranostics. To address this issue, adapters have been introduced; nevertheless, the unstable, non-covalent adapter-vector complex may decrease its efficacy.

In this regard, in a study conducted in 2006, the avidin-biotin system, as one of the strongest non-covalent protein–ligand interactions, was used to redirect Ad5 to dendritic cells. The biotin acceptor peptide (BAP) was genetically incorporated into the fiber protein to generate an Ad-BAP fusion. Because of its high-affinity interaction (10^−15^ M), this system demonstrated high potential for *in vivo* applications, although toxicity was still probable due to the presence of free biotin in the circulation (Maguire et al., 2006). Additionally, in another study, the BAP system was used to compare retargeting of Ad vector through modification of fiber, protein IX, and hexon toward various cell types. In contrast to protein IX and hexon, only fiber modification with high-affinity receptor binding ligands could lead to effective Ad retargeting, most probably due to atypical virus trafficking in case of protein IX and hexon modifications (Campos and Barry, 2006).

In the present study, the bacterial superglue, SpyTag-SpyCatcher, which has been shown to have a median breakage force more than 20 times stronger than the avidin-biotin interaction, was employed (Zakeri et al., 2012; Veggiani et al., 2014). In this system, in contrast to chemical conjugation-based techniques, the SpyTag and SpyCatcher can easily react with one another to form a stable covalent bond under various conditions. The SpyTag can also react at either the N-terminus, C-terminus, or an internal site of protein, making it more flexible than previous split protein-based systems.

In this study, a modified Ad5 vector was generated by inserting the SpyTag peptide into the HI loop of Ad5 fiber knob. The SpyTag on the knob domain acted as an anchoring site for a cell-binding protein (CBP) linked with the SpyCatcher. According to previous studies, any change in the knob can lead to fiber instability; therefore, the structure of the Ad5 fiber knob protein poses a limitation for fiber-modified Ad vector development (Dmitriev et al., 1998). However, considering the rescue of fiber-modified Ad5 containing SpyTag with an infectious unit similar to the control virus with an intact fiber, the insertion of SpyTag into the fiber knob did not affect virus packaging or propagation in our system.

To evaluate the functionality of fmAd5-GFP as a fiber-modified SpyTag-containing Ad5, the CHO-Spy cells with SpyCatcherΔ on their surface were treated with the modified virus. Following SDS-PAGE and Western blotting analysis, which confirmed the ligation ability of fmAd5-GFP with the SpyCatcher (Figure 2B), a comparison of the transduction rate of CHO-Spy *versus* CHO-K1 cells revealed that fmAd5-GFP could bind to CHO-Spy cells and transduce them almost 9–10 times more than CHO-K1 (Figure 4B). Additionally, a 2-5fold increase in the fluorescence intensity of CHO-Spy cells transducted with fmAd5-GFP (relative to CHO-K1) confirmed the efficient binding of SpyCatcher on the CHO-Spy surface with SpyTag on the modified virus (Figure 4A).

The fmAd5-GFP detargeting was examined by application of SpyCatcher-ligated modified virus on A549 as a high CAR-expressing cell line. The significant inhibition of CAR-mediated viral transduction (Figure 5) approves a hypothesis which proposes that Ad5 is remarkably less capable of transducing cells through CARs after binding with an adapter molecule, conjugated to the SpyCatcher (Waehler, et al., 2007). This observation is also comparable to the findings of a study by Dreier et al. (2013) which showed that binding of 1D3nc SHP1 (trimeric DARPins grabbing the knob from three sides) to the knob blocked all CAR-binding sites and completely impaired gene transfer into HEK-293 cells. Nevertheless, due to the uncertainty of SpyCatcher attachment to all three monomers of the knob, the degree of CAR-binding ablation was lower than the trimeric DARPin (1D3nc SHP), which could bind more firmly to all three knob monomers. Generally, non-specific transduction, particularly in hepatocytes, significantly decreased by preventing CAR binding in the modified SpyCatcher-ligated virus.

To retarget fmAd5-GFP, SpyCatcher fused to a nanobody against VEGFR2 was used in the current study. VEGFR2 has been widely targeted for the anti-angiogenic treatment of tumors, as well as diagnosis of various cancers, such as breast and gastric cancers (Masuda et al., 2012; Lian et al., 2019; Masłowska et al., 2021). Currently, camelid nanobody-based therapeutics are being evaluated in clinical trials against various diseases, including cancers. Immunogenicity is one of the main challenges in the application of antibodies, especially when repeated injections are required. Overall, nanobodies exhibit low immunogenicity owing to their high degree of homology with the human VH domain, which can strongly mitigate the potential negative consequences (Jovčevska and Muyldermans, 2020).

Additionally, the single-domain characteristic of nanobodies facilitates their genetic manipulation, allowing for the construction of multivalent nanobodies or their fusion with other proteins. Besides, due to the production of nanobodies using low-cost expression systems, such as *E. coli,* they are appealing tools for a wide range of applications (De Vlieger et al., 2018). Therefore, in this study, we decorated fmAd5-GFP with a SpyCatcher-fused nanobody specific to VEGFR2 as a model CBP in an adapter structure. There was a significant difference in the transduction rate when a high adapter concentration or adapter-to-fiber ratio was used (Figure 6), which is consistent with earlier studies (Dreier et al., 2011). Previously, it was reported that chelating the trimeric knob by bivalent or trivalent adapters could improve the Ad retargeting specificity and efficacy at lower adapter concentrations or adapter-to-fiber ratios (Dreier et al., 2011). Accordingly, in future studies, a triple SpyCatcher adapter can be created using a trimerization motif to achieve optimal retargeting and detargeting at lower adapter concentrations or adapter-to-fiber ratios.

In conclusion, in the present study, using the SpyTag-SpyCatcher protein ligation chemistry, a readily modifiable Ad-based bionanoparticle was developed *in vitro* for retargeting, without any need for genetic manipulation of the viral vector. The results revealed that the insertion of SpyTag peptide into the HI loop of the Ad5 fiber knob did not impair the viral production process; it also did not impair the SpyTag-incorporated knob availability to bind to the adapter molecule. The modified Ad vector was significantly detargeted from its natural CAR and retargeted to VEGFR2. Although VEGFR2 was targeted as a CBP model, this viral vector could be easily modified by covalent binding to target other ligands for various theranostic applications, while significantly mitigating the side effects of systemic Ad administration, including hepatotoxicity.

## Data Availability

The original contributions presented in the study are included in the article/Supplementary Materials, and further inquiries can be directed to the corresponding authors.
