# Supplementary material for: Fiber manipulation and post-assembly nanobody conjugation for adenoviral vector retargeting through SpyTag-SpyCatcher protein ligation
Source: Front Mol Biosci. 2022 Dec 5;9:1039324. doi: 10.3389/fmolb.2022.1039324 (PMC9760943; doi:10.3389/fmolb.2022.1039324)
Supplement: Supplementary file 1 [file Table1.docx]

Table S1: Advantages and disadvantages of various nanovector-based theranostic agents

| Type of Nanovectors | Advantages | Disadvantages | References |
| --- | --- | --- | --- |
| Gold nanoparticles | Multimodal applications (targeting, diagnostics, and therapy)  Low hydrodynamic mean size  adaptability  Stability  low toxicity | High costs for large-scale production  Lack of standard protocols for translation into the clinics  Nonbiodegradability | (Arvizo, Bhattacharya and Mukherjee, 2010) |
| Dendrimers | very precise size and shape controllability  high water solubility  biocompatibility  polyvalency  elicit negligible immune response | Nonspecific cytotoxicity  Limited release of the associated bioactives  Rapid clearance | (Madaan *et al.*, 2014) |
| Quantum dots | High photo- and chemical stability  Potential for synergistic application in diagnostics and therapeutic applications.  simple functionalization | Toxicity concern  Particle aggregation and removal  Air-sensitive | (Zhao and Zhu, 2016) |
| Carbon nanotubes | Large surface area  Ultrahigh functionalization and loading capacities  High penetration capacity to biological barriers  Scaffold for additional agents | Nonbiodegradable  Toxicity (oxidative stress)  Poor aqueous solubility  High production cost | (Riley and Vermerris, 2017) |
| Liposomes | ease of conjugation and functionalization with targeting agents  Rapid cellular uptake and well-characterized cell internalization mechanism  Low immunogenicity  Biocompatibility  Flexibility of synthesis, modification, and formulation | Batch-to-batch variation in manufacturing  Low solubility  High production cost  Limited instability and leakage of loaded materials | (Muthu *et al.*, 2014) |
| Virus-based nanoparticles  (such as adenoviral vectors) | Biocompatibility  Rapid cellular uptake and well-characterized cell internalization mechanism  Self-assembling capability  Endosomal escape activity  High delivery efficacy | High Immunogenicity  Possibility of virus genome integration into host cells (adenoviral vectors are safe in this regard) | (Butt *et al.*, 2022;  Somiya, Liu and Kuroda, 2017) |
